# Multi-Locus Sequence Analysis Reveals Profound Genetic Diversity among Isolates of the Human Pathogen *Bartonella bacilliformis*

**DOI:** 10.1371/journal.pntd.0001248

**Published:** 2011-07-19

**Authors:** Gemma L. Chaloner, Palmira Ventosilla, Richard J. Birtles

**Affiliations:** 1 Institute of Infection and Global Health, University of Liverpool, Liverpool, United Kingdom; 2 Instituto de Medicina Tropical Alexander Von Humboldt, Universidad Peruana Cayetano Heredia, Lima, Peru; 3 School of Environment and Life Sciences, University of Salford, Salford, United Kingdom; Institut Pasteur, France

## Abstract

*Bartonella bacilliformis* is the aetiological agent of human bartonellosis, a potentially life threatening infection of significant public health concern in the Andean region of South America. Human bartonellosis has long been recognised in the region but a recent upsurge in the number of cases of the disease and an apparent expansion of its geographical distribution have re-emphasized its contemporary medical importance. Here, we describe the development of a multi-locus sequence typing (MLST) scheme for *B. bacilliformis* and its application to an archive of 43 isolates collected from patients across Peru. MLST identified eight sequence types among these isolates and the delineation of these was generally congruent with those of the previously described typing scheme. Phylogenetic analysis based on concatenated sequence data derived from MLST loci revealed that seven of the eight sequence types were closely related to one another; however, one sequence type, ST8, exhibited profound evolutionary divergence from the others. The extent of this divergence was akin to that observed between other members of the *Bartonella* genus, suggesting that ST8 strains may be better considered as members of a novel *Bartonella* genospecies.

## Author Summary

Bartonellosis (Carrion's disease) is caused by the bacterium *Bartonella bacilliformis* and is encountered across the Andean cordillera, particularly in Peru. *B. bacilliformis* is transmitted by arthropods, probably sandflies, and exploits humans as a reservoir host, establishing chronic infections characterized by intra-erythrocytic bacteremia. It this thought that asymptomatic infections are common among the residents of bartonellosis-endemic regions. However, several thousand cases of *B. bacilliformis*-induced severe haemolytic anaemia are reported each year, frequently among visitors to endemic regions but also among inhabitants of an increasing number of new foci of disease. To better understand the epidemiology of bartonellosis, a clearer understanding of how *B. bacilliformis* strains circulating in endemic regions and in new foci are related to one another is required. Here, we describe the development and application of a new genotyping tool for addressing this shortfall, namely multilocus sequence typing (MLST). We applied MLST to 43 *B. bacilliformis* isolates of varying provenance, delineating eight distinct genotypes, some of which had obvious epidemiological correlates. Surprisingly, one genotype exhibited profound divergence from the other seven, and the extent of this divergence suggested that some bartonellosis may be caused by a *Bartonella* species specifically related to, yet distinct from, *B. bacilliformis*.

## Introduction

Bartonellosis, or Carrion's disease, caused by the bacterium *Bartonella bacilliformis*, has long been recognised in the Andean region of South America, particularly in the high valleys lining the western side of the cordillera in central Peru [1]. Bartonellosis may present in two markedly different clinical manifestations. Firstly, Oroya fever is characterised by fever, headache, pallor and myalgia, which progresses to a severe haemolytic anaemia. Mortality rates as high as 88% have been described in untreated patients with this manifestation. Alternatively, infection may provoke “verruga peruana” characterised by angiogenic skin lesions akin to bacillary angiomatosis caused by *Bartonella henselae* and *Bartonella quintana*. Although the appearance of lesions may be dramatic, verruga peruana tends to be self-limiting and not life-threatening. The natural cycles of *Bartonella* species are characterised by mammalian reservoirs and arthropod vectors, and for *B. bacilliformis*, humans appear to be the sole reservoir host and sandflies (*Lutzomyia* spp.) are considered the most likely vectors. Asymptomatic and chronic infections of people living in areas where *B. bacilliformis* is endemic are thought to be common [2], [3].

Monitoring of bartonellosis in Peru over the past two decades has revealed some dramatic epidemiological changes. The number of cases collated nationally by the Instituto Nacional de Salud rose from about 3,000 per annum in the 1990s to over 10,000 per annum between 2004 and 2006, before declining again over the past four years (http://www.ins.gob.pe/portal), and numerous new foci of bartonellosis have been identified in regions of the country where the disease was previously unknown [2], [4]–[7]. The disease has also been reported in new locales in Colombia and Ecuador [5], [8], [9]. The ecological or anthropological bases for these changes are unknown, although it has been postulated that they may have resulted from warmer, wetter weather provoking increases in the population size and range of sandfly vectors [10].

Only very few studies exploring the genetic diversity of *B. bacilliformis* have been published [11], [12]. These efforts employed a variety of different typing methods including pan-genomic approaches such as amplified fragment length polymorphism and infrequent restriction site polymerase chain reaction (PCR), and/or comparison of nucleotide sequence variation at loci including the citrate synthase gene (*gltA*), invasion-associated locus B gene (*ialB*) and, most frequently, the 16S-23S rDNA intergenic spacer region (ISR). All these approaches have delineated genotypes within the species, and have been useful in characterising the molecular epidemiology of bartonellosis [11], [12].

Multi-locus sequence typing (MLST) is now established as a powerful approach to defining the population structures of bacterial species and to explore the evolutionary mechanisms that have shaped these population structures [13]–[15]. MLST schemes for *B. henselae* and *B. quintana* have already been described and have proven to be of value [16]–[21]. The aim of the present study was to develop a MLST scheme for *B. bacilliformis* then to exploit the scheme to explore the population structure of the species using a representative archive of isolates obtained from patients presenting with different infection manifestations and from different regions of Peru.

## Materials and Methods

### Terminology

The following definitions of the terms isolate and strain proposed by Struelens and colleagues [22] are used throughout the text: “isolate,” a population of microbial cells in pure culture derived from a single colony on an isolation plate and characterized by identification to the species level; “strain,” an isolate or group of isolates exhibiting phenotypic and/or genotypic traits which are distinctive from those of other isolates of the same species.

### *B. bacilliformis* isolates and growth conditions

A total of 43 isolates of *B. bacilliformis* were included in the study. The sources of these isolates, together with their date of isolation, are presented in Table 1. Most of the isolates in this study were isolated and/or archived by one of us (PV). The exceptions were the isolates with an NCTC prefix, which came from the National Collection of Type Cultures, and the isolates CON600-1 and Cond044, which were kindly provided, by Larry Laughlin and Judith Chamberlin of the Uniformed Services University of the Health Sciences, Bethesda, Md. Isolates were grown on Columbia agar plates (Columbia Agar base, Oxoid) supplemented with 10% defibrinated horse blood and incubated at 30°C.

**Table 1:** Characteristics of, and MLST data for, the 43 *B. bacilliformis* isolates studied.

isolate designation	geographic location where infection was acquired (endemic/new focus)	year	age/sex	disease	MLST allelic profile							ST
					*ftsZ*	*flaA*	*ribC*	*rnpB*	*rpoB*	*bvrR*	*groEL*	
KC583	Huarochiri, Lima (End)	1960s	NK	NK	1	1	1	1	1	1	1	1
KC584	Churcampa, Huancavelia (End)	1960s	NK	NK	1	1	1	1	1	1	1	1
T2	Huaraz, Ancash (End)	1999	NK	OF	1	1	1	1	1	1	1	1
Hua-Rub	Huarochiri, Lima (End)	1999	23 M	OF	1	1	1	1	1	1	1	1
Sih-Ism	Sihuas, Ancash (End)	1999	28 M	OF	1	1	1	1	1	1	1	1
Hua-Chu	Huarochiri-Quiripa, Lima (End)	1999	16 M	OF	1	1	1	1	1	1	1	1
Hua-Mar	Huarochiri-Puellucanchi-Lima (End)	1999	2 M	OF	1	1	1	1	1	1	1	1
Hua-Nol	Huarochiri-Quinti, Lima (End)	1999	50 M	OF	1	1	1	1	1	1	1	1
Alca	Caraz-Ancash- (End)	1999	17 M	OF	1	1	1	1	1	1	1	1
Cas	Caraz-Ancash (End)	1999	43 M	AS	1	1	1	1	1	1	1	1
Quillay	Huaraz, Ancash (End)	2003	NK	NK	1	1	1	1	1	1	1	1
Quispe	Huanuco -Huacrachucro-(end)	2002	33M	OF	1	1	1	1	1	1	1	1
Fili	Yungay, Ancash (End)	2004	2 M	NK	1	1	1	1	1	1	1	1
Vega	Comas, Lima (End)	2005	17 M	NK	1	1	1	1	1	1	1	1
Sot	Huaraz, Ancash (End)	2005	47 M	NK	1	1	1	1	1	1	1	1
Bon	San Martin (NK)	2006	23 M	NK	1	1	1	1	1	1	1	1
DB06-P154	Canete, Lima (NK)	2006	11 M	OF	1	1	1	1	1	1	1	1
DB07-P219	Huaral, Lima (NK)	2007	26 M	OF	1	1	1	1	1	1	1	1
DB07-P207	Huaral, Lima (NK)	2007	14 M	OF	1	1	1	1	1	1	1	1
FBC-220	Huaraz, Ancash (End)	NK	NK	NK	1	1	1	1	1	1	1	1
CONDO44	Huaylas, Ancash (End)	1997	10 F	AS	2	3	2	2	2	1	1	2
NCTC12134	NK	1949	NK	NK	1	2	2	3	1	2	2	3
NCTC12135	NK	1941	NK	NK	1	2	2	3	1	2	2	3
CON600-1	Huaylas, Ancash (End)	1997	8 F	AS	1	2	2	3	1	3	2	4
Olivares	Llumpe, Ancash (End)	2003	5 M	NK	1	2	2	3	1	3	2	4
Luna	Chimbote, Ancash (End)	2003	17 M	NK	1	2	2	3	1	3	2	4
Mul	NK	2005	NK	NK	1	2	2	3	1	3	2	4
DB06-P162	Huarochiri, Lima (End)	2006	NK M	NK	1	2	2	3	1	3	2	4
FBC-186	Huaraz, Ancash (End)	NK	NK	NK	1	2	2	3	1	3	2	4
FBC-196	Huaraz, Ancash (End)	NK	NK	NK	1	2	2	3	1	3	2	4
Mor	Ancash (NK)	NK	NK	NK	1	2	2	3	1	3	2	4
150-01	Huaraz, Ancash (End)	NK	NK	NK	1	2	2	3	1	3	2	4
Agui	Huarochiri, Lima (End)	NK	NK	NK	1	2	2	3	1	3	2	4
Gan	NK	NK	NK	NK	1	2	2	3	1	3	2	4
CUSCO5	Urubamba, Cusco (NF)	1998	5 M	OF	3	1	1	1	1	4	1	5
CUSCO407	Urubamba, Cusco (NF)	1998	NK	AS	3	1	1	1	1	4	1	5
Cusco-Ana	Urubamba, Cusco (NF)	1998	10 F	OF	3	1	1	1	1	4	1	5
CUSCO8	Urubamba-Pallata, Cusco (NF)	1998	28 M	OF	3	1	1	1	1	4	1	5
ER-Cha	Luya, Amazonas (NF)	1999	16 M	OF	3	1	1	4	1	5	1	6
ER-Yal	Luya, Amazonas (NF)	1999	NK	OF	3	1	1	4	1	4	1	7
ER-Tej	Luya, Amazonas (NF)	1999	38 M	OF	3	1	1	4	1	4	1	7
LA6.3	Bolognesi, Ancash (End)	1990	NK	OF	4	4	3	5	3	6	3	8
Luc-Uba	Mariscal Luzuriaga, Ancash (End)	1999	4 F	OF	4	4	3	5	3	6	3	8

NF  =  New foci, End  =  Endemic, NK  =  Not Known, OF  =  Oroya Fever, AS  =  Asymptomatic.

### MLST

Internal fragments of approximately 300 to 500 base pairs (bp) were amplified from each of the seven genetic loci and evaluated for use in the MLST scheme (Table 2). A sweep of colonies from each plate was harvested into sterile, distilled water and boiled at 100°C for 10 minutes for use as template in PCRs.

**Table 2:** Primers used for the amplification and sequencing of the seven loci evaluated for the *B. bacilliformis* MLST scheme.

locus	putative gene product	product size (bp)	position^1^	forward primer (5′-3′)	reverse primer (5′–3′)	reference
*bvrR*	regulatory protein	486	1385452–1385937	GACCGCAATATTTTGACATC	GCATCCATCAAAGCATCACGACTT	[16]
*ribC*	riboflavin synthase alpha subunit	349	652816–653164	GATATCGGTTGTGTTGAAGA	AAAGGCGCTAACTGTTC	[20]
*ftsZ*	cell division protein	497	969686–970182	CTCAAGTAGGAGTGCTGCTA	CCAATTGATCTTCCTCGTTTAC	this study
*groEL*	heat shock protein	442	1211811–1212252	CAACAGAAGTTGAAGTGAAAG	TAGAAATCCATTCCGCCCATT	this study
*flaA*	flagellin A	517	1076953–1077409	TTCACTGAAGCTGCTGATAAA	CTTGTATTTGTAACGTCGTA	this study
*rnpB*	RNA subunit of endoribonuclease RNase P	297	988378–988674	CGGGATCCGGGGAGGAAAGTCCGGGC	CGGAATTCRTAAGCCGGRTTCTGT	[21]
*rpoB*	RNA polymerase beta subunit	363	579639–580001	ACGCCTGAAGGTCCAAATAT	CTTCAGAACGGATCAATGGA	this study

1 Corresponding to the complete genome sequence of *B. bacilliformis* strain KC583 Genbank accession number CP000524.

Primers for amplification and sequencing of MLST loci were designed using Primer3 software (Table 2). The primers for the loci *bvrR*, *ribC* and *rnpB* have been previously described [18], [23], [24]. Reaction mixtures (25 µl) contained the following: 12.5 µl of 2xPCR mastermix (Abgene), 0.5 µl of a 20 ρmol µl-1 solution of both forward and reverse primers, 10.5 µl sterile, distilled water and 1 µl of DNA template. Reaction mixtures were exposed to a thermal cycle consisting of denaturation at 96°C for 5 min followed by 40 cycles of 96°C for 10 sec, 55°C for 10 sec and 72°C for 50 sec, with a final extension step of 72°C for 10 minutes. To verify the specificity of newly-designed primers, amplification products were electrophoretically resolved on 1% agarose gels then visualized using UV light following staining with ethidium bromide. The nucleotide base sequences of both strands of amplification products were determined commercially.

### Analysis of MLST data

The nucleotide sequences were analysed with Chromas Pro software V 1.4.1 (http://www.technelysium.com.au/ChromasPro.html) and alignments carried out using MEGA V4.0 [25]. For every locus, alleles were assigned a number according to the order in which they were encountered (although alleles possessed by the *B. bacilliformis* type strain, KC583, were always assigned allele number 1 for each loci examined). For each isolate, the combination of alleles at each loci examined (the allelic profile) defined the sequence type (ST), and these were assigned numbers in order of encounter.

Relatedness among *B. bacilliformis* isolates was inferred by comparison of both allelic profiles and concatenated sequence data using START version 2 [26]. The phi test and splits decomposition analysis which are used to assess conflict in phylogenetic signal from different loci were carried out using the default settings in Splitstree4 [27].

### Nucleotide sequence accession numbers

Newly encountered alleles have been submitted to Genbank under the following accession numbers JF326267 to JF326294.

## Results

### MLST and phylogeny

**Figure 1:**
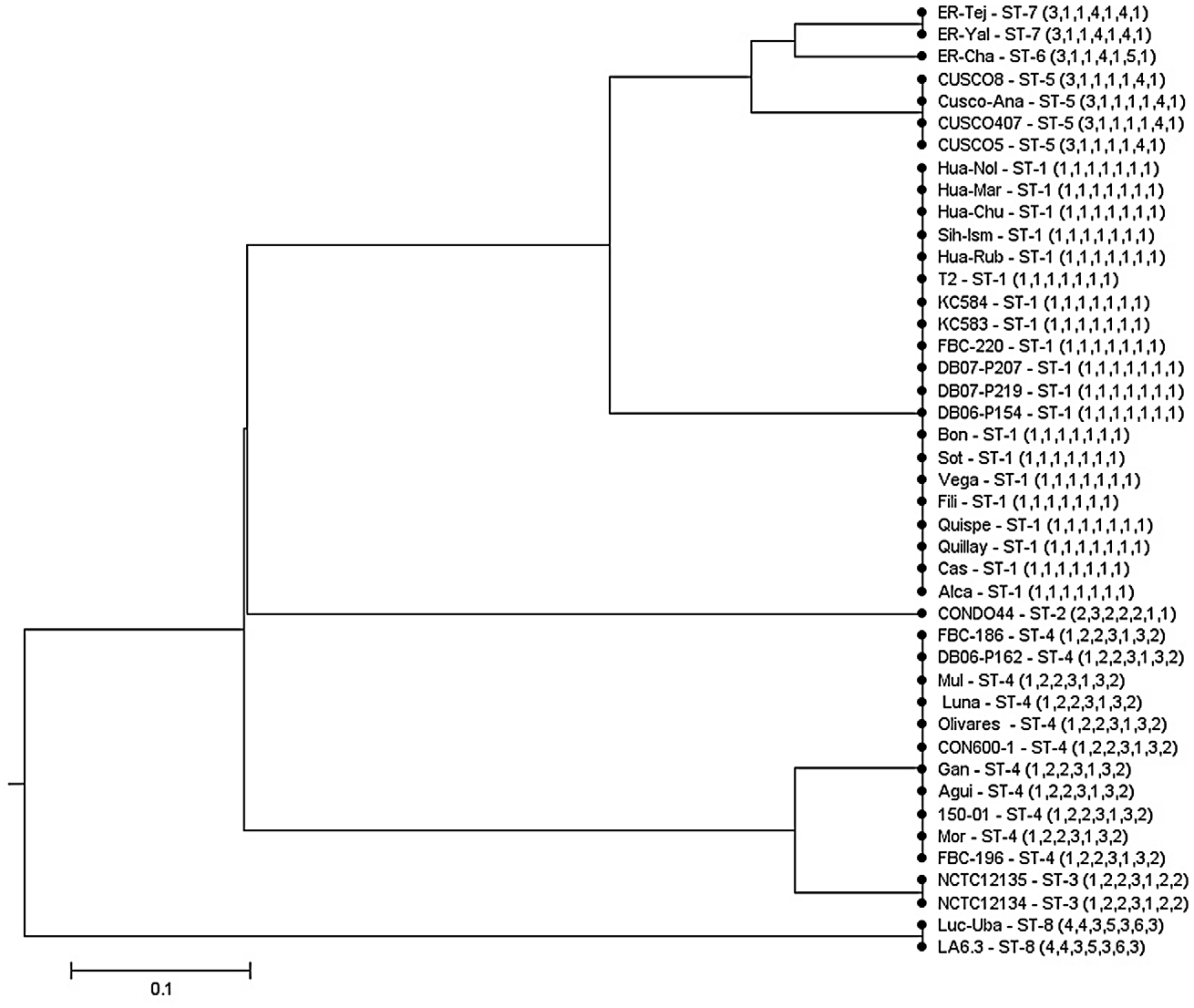
Dendrogram of the 43 isolates of *B. bacilliformis* as constructed by UPGMA cluster analysis of MLST data.

Sequence data were obtained for the seven genetic loci from all 43 isolates included in the study. The length of sequence data obtained at each locus ranged from 297 bp to 517 bp (Table 2). Comparison of sequence data revealed variation at all loci, with dissimilarity ranging from 3.0 % (*rpoB*) to 8.2 % (*ftsZ*) (Table 3). The number of alleles for each locus ranged from 3 to 6 (Table 3). Comparison of allelic profiles revealed eight sequence types (STs). ST1 was encountered most frequently, with 20 isolates possessing this ST. Eleven isolates belonged to ST4. Two STs, ST2 and ST6 were represented by only one isolate each. A dendrogram of relatedness, generated by UPGMA cluster analysis of the allelic profiles of 43 *B. bacilliformis* isolates, was generated (Figure 1). In this dendrogram, the six of the eight STs were resolved into two clonal complexes (i.e. ST sharing 4 or more alleles), one containing ST1, ST5, ST6 and ST7, and the second containing ST3 and ST4. ST7 was found to be a single locus variant of ST5 and ST6, and ST3 and ST4 were also found to be single locus variants of one another. ST2 shared, at most, only two alleles with other STs, whereas ST8 was found to possess unique alleles at all seven loci (Figure 1).

**Table 3:** Characteristics of the seven loci evaluated for the *B. bacilliformis* MLST scheme.

locus	isolates belonging to STs 1-8		isolates belonging to STs 1-7	
	number of alleles	number (%) of variable sites	number of alleles	number (%) of variable sites
*ftsZ*	4	41 (8.2)	3	5 (1.0)
*flaA*	4	32 (6.2)	3	12 (2.3)
*ribC*	3	17 (4.9)	2	3 (0.9)
*rnpB*	5	10 (3.4)	4	3 (1.0)
*rpoB*	3	11 (3.0)	2	5 (1.4)
*bvrR*	6	19 (3.9)	5	5 (1.0)
*groEL*	3	27 (6.1)	2	4 (0.9)
all loci	-	157 (5.3)	-	37 (1.3)

**Figure 2:**
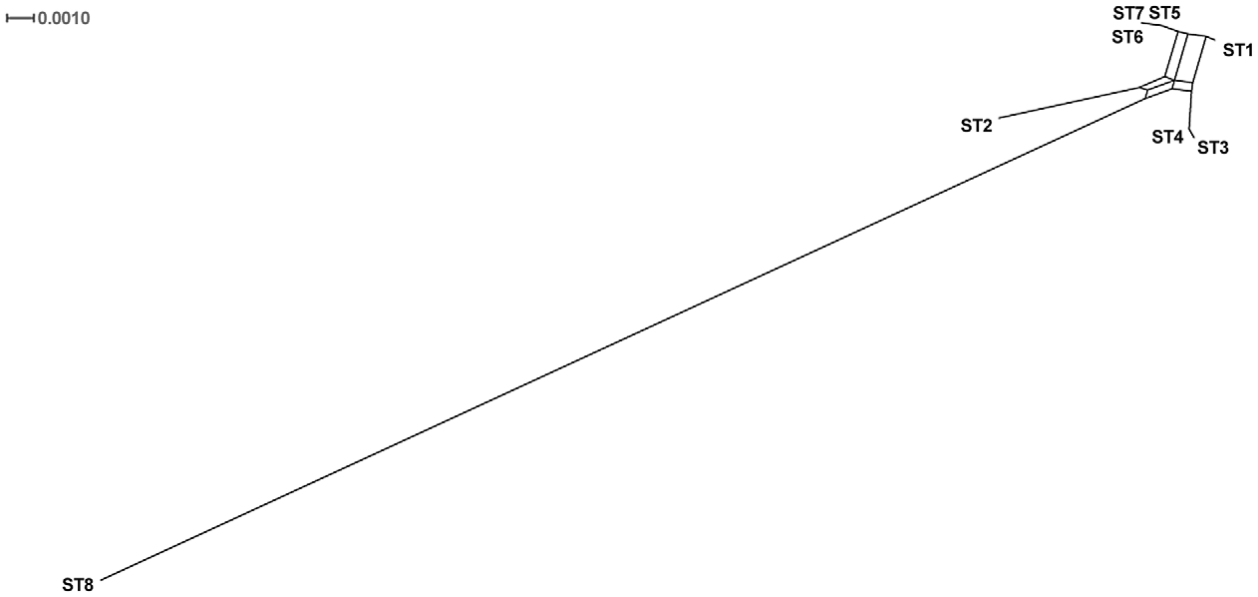
Splits decomposition was used to detect evidence for a past history of recombination in the sequences. The extensive reticulation suggests that recombination has occurred relatively frequently. However, ST 8 remains distinct.

Sequence comparison not only confirmed ST8 to be outlying to the other *B. bacilliformis* STs, but also demonstrated the extent of dissimilarity between this and other STs (Table 3). Among STs 1–7, only 37 variable positions (1.3%) among the 2951 bp of sequence data were observed, whereas among STs 1–8 this number rose to 157 (5.3%) (Table 3). Phylogenetic analysis, inferred from alignment of concatenated sequence data for all seven loci (2951 bp) confirmed the profound divergence of isolates belonging to ST8 from all other *B. bacilliformis* isolates studied (Figure 2). This dendrogram, inferred using splits decomposition analysis, is also characterised by a network structure that suggests recombination has influenced the divergence of *B. bacilliformis* STs. This suggestion is supported by the results of a phi test, which also indicated significant evidence for recombination (P = 0.042).

**Figure 3:**
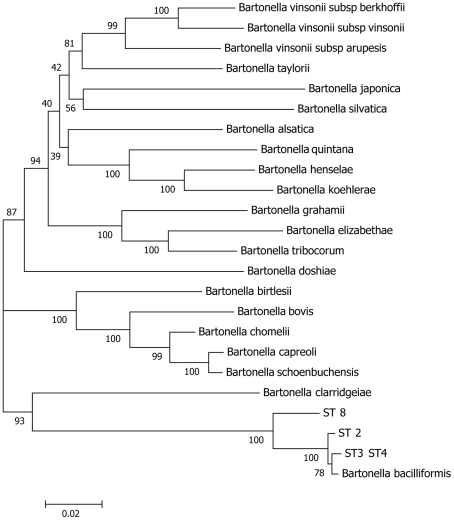
Phylogenetic relationship of species of the genus Bartonella inferred from concatenated sequences of *ribC rpoB groEL* and *gltA* fragments. The phylogenetic tree was constructed using the NJ method. Using these fragments *B. bacilliformis* ST1, ST5, ST6 and ST8 are identical to the *B. bacilliformis* type strain. Bootstrap values (percentages of 1000 replications) above 70% are indicated at the nodes.

We further explored the extent of divergence between ST8 and other *B. bacilliformis* sequence types by assessing the phylogenetic distance between them relative to inter-species divergence across the *Bartonella* genus. We assembled concatenated sequences from *ftsZ, gltA, groEL, ribC* and *rpoB* data available for all valid *Bartonella* species, and inferred phylogeny from a 1323 bp alignment of these sequences (Figure 3). These data suggest that the divergence observed between ST8 and other *B. bacilliformis* STs is as great as, and occasionally exceeds, that separating *Bartonella* species.

### Molecular epidemiology

All isolates belonging to ST1, ST2, ST3 and ST4 were obtained from patients living in the region of Peru where bartonellosis has long been considered endemic. ST5 comprised solely of isolates associated with a large outbreak of bartonellosis in Urubamba, Cusco, in 1999. The three ST6 and ST7 isolates were associated with a new focus of bartonellosis in Pisuquia, Amazonas. The two ST8 isolates were obtained from patients living in the same region of Peru where ST1 to 4 were encountered.

Isolates belonging to ST1 were collected as long ago as the “early” 1960s and as recently as 2007, suggesting its continued circulation in the “endemic region” of Peru for at least 40 years. One of the four isolates obtained from the asymptomatic patients was the only member of ST2, however the other three isolates from asymptomatic patients belonged to STs that also included isolates obtained from patients with overt disease (Oroya fever). For 20 patients, no information about their disease manifestation was available.

## Discussion

*B. bacilliformis* remains an enigmatic pathogen; despite being identified over 100 years ago and continuing to pose a significant public health threat, our knowledge of its ecology and pathogenicity, and the epidemiology of the infections it causes, remains very incomplete. This shortfall is particularly unsatisfactory as, given its limited geographic distribution and its apparent specific adaptation to humans, eradication of *B. bacilliformis* infections using vaccination should be a realistic goal. Perhaps the most significant finding of the current study is that *B. bacilliformis* may not be a single species; we use MLST data to provide clear evidence that a minority of isolates recovered from patients with haemolytic anaemia (Oroya fever) have diverged from other *B. bacilliformis* isolates to a degree akin to that observed between other *Bartonella* species. These isolates, belonging to ST8 in our study, are therefore likely to belong to a novel *Bartonella* genospecies, although further (polyphasic) characterization of these isolates is needed to support their formal taxonomic reclassification. The two ST8 isolates were not epidemiologically linked, being obtained nine years apart, and from locations that lie 150 km from one another. Two further isolates that are also potential members of ST8/a new genospecies have been described elsewhere [28]; the partial *gltA* and ISR sequences of these isolates are indistinguishable from those of LA6.3 and Luc-Uba, the two ST8 isolates included in the current study. The two further isolates were both obtained from Caraz, a town in Ancash where bartonellosis has long been recognized, which lies at the heart of the region where bartonellosis is considered endemic [28]. At present, we have no insight into the ecological basis for the divergence of ST8 from other *B. bacilliformis* STs; on a broad geographical scale at least, the distribution of ST8 overlaps with those of STs1-4. To what extent the genetic divergence of ST8 from other *B. bacilliformis* STs is reflected in phenotypic differences is, as yet, unclear; the growth requirements for ST8 isolates are, apparently indistinguishable from those of other *B. bacilliformis* strains (i.e, unusually for *Bartonella* species, they grow at 30°C in the absence of CO_2_), and their colonial and microscopic morphology is the same. Furthermore, antiserum from a patient infected with a “likely ST8” strain (Vega) reacted strongly with antigens prepared from other *B. bacilliformis* isolates including EC-01, an isolate which bore genotypic similarity to ST1 strains [28]. These shared phenotypic traits are significant from a diagnostic perspective as laboratory confirmation of infection status relies primarily on microscopic examination of blood smears, bacterial isolation and, albeit less frequently, demonstration of specific antibodies [2]–[4], [11], [28]. These approaches would appear to be as suitable for ST8 strains as for less genetically divergent *B. bacilliformis* strains.

In general, the delineation of *B. bacilliformis* using MLST matches that inferred using other typing methods. MLST-defined STs are akin to the genotypes identified in an earlier study on the basis of AFLP analysis and comparison of ISR sequences. That MLST has delineated more genotypes than either AFLP or ISR-based typing is the result of MLST, but not other schemes, differentiating between strains associated with a new focus of bartonellosis in Luya province, and segregating Cond044 from ST1 strains. The first of these differences was the result of a single nucleotide polymorphism in the *bvrR* locus, and was verified by repeat amplification and sequencing of this locus. Thus, even in locations where bartonellosis has only recently been recognized, genotypically distinct strains are in circulation. The second of these differences is also noteworthy as the allelic profile of Cond044 was very different (only 2/7 shared alleles) from that of ST1, with which it clustered using AFLP and ISR-based typing [11]. Indeed, among the non-ST8 sequence types, ST2, of which Cond044 was the sole representative, was the most divergent. Thus there appears to be marked incongruence between MLST and an approach involving AFLP and ISR sequence comparison in determining the position of Cond044 within the genotypic spectrum of the species. Closer re-examination of AFLP data suggests that although Condo44 clustered with ST1 strains, it was the outlier of the cluster, however its ISR sequence was indistinguishable from that of ST1 isolates. Amongst the MLST loci, ST1 and ST2 shared the same *bvrR* and *groEL* alleles, and sequence dissimilarity at the other five loci ranged from 0.7% (*rnpB*) to 2.3% (*flaA*), emphasizing the existence of markedly different levels at variation in different parts of the genome and hence the benefits of using MLST approach.

Complete genome sequences are currently available for six *Bartonella* species, *B. henselae, B. quintana, B. tribocorum, B. bacilliformis*, *B. grahamii and B. clarridgeiae* [29]–[32], with more in draft [32]. Comparative analysis of four of the complete genome sequences has revealed that diversity between them is primarily shaped by significant expansions (due to lateral gene transfer and gene duplication) and reductions (due to gene decay and deletion) in their accessory genomes [33]. Recently, high recombination frequencies and large variations in genome size have been reported in *B. grahamii* [34], and recombination has been identified as playing a dominant role in the diversification of four rodent-associated *Bartonella* species [35]. Our analysis suggests that recombination has also had a strong influence in shaping.

*B. bacilliformis* genomes. Although splitstree analysis suggested that the extent of recombination between STs was greater than that reported for *B. henselae* [18], quantification of the relative rate of recombination in *B. bacilliformis* compared to other *Bartonella* genomes was not attempted.

MLST analysis on our isolate archive confirmed the earlier observation, based on AFLP and ISR sequence comparison, that genotypes associated with new foci of bartonellosis were distinct from those present in the region where bartonellosis is considered endemic, with STs 5, 6 and 7 being encountered only outside this region. This finding contrasts with work reported by others in which such geographic delineation of *B. bacilliformis* isolates was not observed. Hambuch and colleagues (2004) used infrequent restriction site PCR in combination with partial *fla* and *ialB* sequence comparison to explore genotypic relationships among isolates from patients in Caraz (endemic region) and isolates associated with the outbreak of disease in Urubamba in 1998 (epidemic) [12]. They did not detect significant differences in variation between the two populations or distinguished one population from the other. Why these results should contrast with those of the current and other previous studies [11] is unclear. However, more recently, Lydy and colleagues (2008) have also reported the presence of isolates similar to those belonging to ST5 (i.e. associated with the Urubamba outbreak) in Caraz and elsewhere in the endemic zone [28]. Thus, it appears increasingly likely that, due to its relatively small size and the opportunistic nature of collection, our archive is limited in terms of its representation of the diversity of genotypes circulating in Peru. This shortfall can only be accurately addressed with systematic surveys, which will require considerable resources, although MLST appears to be an appropriate genotyping method to employ when such a study is instigated.

## References

[1] Herrer A (1990). Epidemiologia de la verruga peruana..

[2] Kosek M, Lavarello R, Gilman RH, Delgado J, Maguina C (2000). Natural history of infection with *Bartonella bacilliformis* in a nonendemic population.. J Infect.

[3] Chamberlin J, Laughlin LW, Romero S, Solorzano N, Gordon S (2002). Epidemiology of endemic *Bartonella bacilliformis*: A prospective cohort study in a Peruvian mountain valley community.. J Infect Dis.

[4] Ellis BA, Rotz LD, Leake JAD, Samalvides F, Bernable J (1999). An outbreak of acute bartonellosis (Oroya fever) in the Urubamba region of Peru, 1998.. Am J Trop Med Hyg.

[5] Huarcaya E, Maguina C, Torres R, Rupay J, Fuentes L (2004). Bartonelosis (Carrion's Disease) in the pediatric population of Peru: an overview and update.. Braz J Infect Dis.

[6] Maco V, Maguina C, Tirado A, Vidal JE (2004). Carrion's disease (Bartonellosis bacilliformis) confirmed by histopathology in the High Forest of Peru.. Rev Inst Med Trop Sao Paulo.

[7] Maguina C, Gotuzzo E (2000). Bartonellosis - New and old. Infect. Dis. Clin.. North Am.

[8] Alexander B (1995). A review of Bartonellosis in Ecuador and Colombia.. Am J Trop Med Hyg.

[9] Cooper P, Guderian R, Paredes W, Daniels R, Perera D (1996). Bartonellosis in Zamora Chinchipe province in Ecuador.. Trans R Soc Trop Med Hyg.

[10] Zhou J, Lau WKM, Laughlin LW, Masuoka PM, Andre RC (2002). The effect of regional climate variability on outbreak of epidemics of bartonellosis in Peru.. Third Symposium on Environmental Applications:Facilitating the Use of Environmental Information.

[11] Birtles RJ, Fry NK, Ventosilla P, Caceres AG, Sanchez E (2002). Identification of *Bartonella bacilliformis* genotypes and their relevance to epidemiological investigations of human bartonellosis.. J Clin Microbiol.

[12] Hambuch TM, Handley SA, Ellis B, Chamberlin J, Romero S (2004). Population genetic analysis of *Bartonella bacilliformis* isolates from areas of Peru where Carrion's disease is endemic and epidemic.. J Clin Microbiol.

[13] Enright MC, Spratt BG (1999). Multilocus sequence typing.. Trends Microbiol.

[14] Maiden MCJ, Bygraves JA, Feil E, Morelli G, Russell JE (1998). Multilocus sequence typing: A portable approach to the identification of clones within populations of pathogenic microorganisms.. Proc Natl Acad Sci U S A.

[15] Platonov AE, Shipulin GA, Platonova OV (2000). Multilocus sequence typing: A new method and the first results in the genotyping of bacteria.. Russ J Genet.

[16] Arvand M, Feil EJ, Giladi M, Boulouis HJ, Viezens J (2007). Multi-Locus Sequence Typing of *Bartonella henselae* Isolates from Three Continents Reveals Hypervirulent and Feline-Associated Clones.. Plos One.

[17] Arvand M, Raoult D, Feil EJ (2010). Multi-Locus Sequence Typing of a Geographically and Temporally Diverse Sample of the Highly Clonal Human Pathogen *Bartonella quintana*.. PLoS One.

[18] Iredell J, Blanckenberg D, Arvand M, Grauling S, Feil EJ (2003). Characterization of the natural population of *Bartonella henselae* by multilocus sequence typing.. J Clin Microbiol.

[19] Lindroos H, Vinnere O, Mira A, Repsilber D, Naslund K (2006). Genome rearrangements, deletions, and amplifications in the natural population of *Bartonella henselae*.. J Bacteriol.

[20] Mietze A, Morick D, Kohler H, Harrus S, Dehio C (2010). Combined MLST and AFLP typing of *Bartonella henselae* isolated from cats reveals new sequence types and suggests clonal evolution.. http://dx.doi.org/10.1016/j.vetmic.2010.08.012.

[21] Yanagihara M, Tsuneoka H, Hoshide S, Ishido E, Umeda A (2010). Molecular typing of *Bartonella henselae* DNA extracted from human clinical specimens and cat isolates in Japan.. FEMS Immunol Med Microbiol.

[22] Struelens MJ (1996). Consensus guidelines for appropriate use and evaluation of microbial epidemiologic typing systems.. Clin Microbiol Infect.

[23] Bereswill S, Hinkelmann S, Kist M, Sander A (1999). Molecular analysis of riboflavin synthesis genes in *Bartonella henselae* and use of the ribC gene for differentiation of *Bartonella* species by PCR.. J Clin Microbiol.

[24] Pitulle C, Strehse C, Brown JW, Breitschwerdt EB (2002). Investigation of the phylogenetic relationships within the genus Bartonella based on comparative sequence analysis of the rnpB gene, 16S rDNA and 23S rDNA.. Int J Syst Evol Microbiol.

[25] Tamura K, Dudley J, Nei M, Kumar S (2007). MEGA4: Molecular evolutionary genetics analysis (MEGA) software version 4.0.. Mol Biol Evol.

[26] Jolley KA, Feil EJ, Chan MS, Maiden MCJ (2001). Sequence type analysis and recombinational tests (START).. Bioinformatics.

[27] Huson DH, Bryant D (2006). Application of phylogenetic networks in evolutionary studies.. Mol Biol Evol.

[28] Lydy SL, Eremeeva ME, Asnis D, Paddock CD, Nicholson WL (2008). Isolation and characterization of *Bartonella bacilliformi*s from an expatriate Ecuadorian.. J Clin Microbiol.

[29] Alsmark CM, Frank AC, Karlberg EO, Legault BA, Ardell DH (2004). The louse-borne human pathogen Bartonella quintana is a genomic derivative of the zoonotic agent *Bartonella henselae.*. Proc Natl Acad Sci U S A.

[30] Saenz HL, Engel P, Stoeckli MC, Lanz C, Raddatz, G (2007). Genomic analysis of *Bartonella* identifies type IV secretion systems as host adaptability factors.. Nat Genet.

[31] Berglund EC, Frank AC, Calteau A, Vinnere Pettersson O, Granberg F (2009). Run-off replication of host-adaptability genes is associated with gene transfer agents in the genome of mouse-infecting *Bartonella grahamii.*. PLoS Genet.

[32] Engel P, Salzburger W, Liesch M, Chang, CC, Maruyama S (2011). Parallel Evolution of a Type IV Secretion System in Radiating Lineages of the Host-Restricted Bacterial Pathogen *Bartonella.*. PLoS Genet.

[33] Engel P, Dehio C, DeReuse H, Bereswill S (2009). Genomics of Host-Restricted Pathogens of the Genus *Bartonella.*. Microbial Pathogenomics.

[34] Berglund EC, Ellegaard K, Granberg F, Xie ZP, Maruyama, S (2010). Rapid diversification by recombination in *Bartonella grahamii* from wild rodents in Asia contrasts with low levels of genomic divergence in Northern Europe and America.. Mol Ecol.

[35] Paziewska A, Harris PD, Zwolinska L, Bajer A, Sinski E (2011). Recombination Within and Between Species of the Alpha Proteobacterium *Bartonella* Infecting Rodents.. Microb Ecol.

